# Supplementary material for: Strategic equity compensation: A delphi-AHP approach to industry-specific governance design for RSUs
Source: PLoS One. 2026 Mar 19;21(3):e0343560. doi: 10.1371/journal.pone.0343560 (PMC13001955; doi:10.1371/journal.pone.0343560)
Supplement: Attachment — Submitted filename: Review_PLOSONE.pdf [file pone.0343560.s003.pdf]

Dear all,

I appreciate the opportunity to learn about your work, entitled "Strategic Equity Compensation: A Delphi-AHP Approach to Industry-Specific RSUs Governance Design" and to contribute in some way to the writing and reporting of this research. I congratulate you on your initiative and commitment to this work.

I recommend publication, provided the following requirements are met:

Introduction

In the first paragraph, you must indicate the source of the information about Microsoft. Even if it is old and not common sense, I suggest using more recent information in this context.
Use references from authors in the field of scientific methodology to support the research justification, in order to meet and demonstrate scientific rigor.

Literature Review

Review the writing format of the cited authors, verify their inclusion in the references, and pay attention to years and spelling.

Updating references. Even if seminal authors are cited, other more recent works may be cited to maintain an up-to-date and recent literature review, covering at least the last 5 years.

Materials and Methods

Identify and characterize the research to improve the reader's understanding and provide references in this regard, highlighting the scientific rigor of the study.
It is important to emphasize the hierarchical structure of the study problem, given that the journal does not restrict the number of pages.

References

Pay attention to the authors cited in the text, spelling, year of publication, and verification of authors cited throughout the text.

Sincerely

Reviser
