# Supplementary material for: Strategic equity compensation: A delphi-AHP approach to industry-specific governance design for RSUs
Source: PLoS One. 2026 Mar 19;21(3):e0343560. doi: 10.1371/journal.pone.0343560 (PMC13001955; doi:10.1371/journal.pone.0343560)
Supplement: Attachment — Submitted filename: Comments to the Authors_PONE-D-25-36569.pdf [file pone.0343560.s004.pdf]

Comments to the Authors:

Manuscript ID: PONE-D-25-36569

Title: Strategic Equity Compensation: A Delphi–AHP Approach to Industry-Specific RSUs Governance Design

Dear Author/s

Thank you for your manuscript to PLOS ONE. After review of the manuscript, the reviewers' reports, and an internal editorial assessment, we find that the manuscript addresses a relevant and underexplored topic: the design of industry-specific RSU governance frameworks using a combined Delphi-AHP approach. This manuscript demonstrates meaningful potential but several substantial revisions are required before the manuscript can be considered for publication. Please find detailed, constructive comments as follows:

- The abstract offers conceptual context but is devoid of critical analytical (data) evidence to substantiate the assertions. Also, limitations like small sample sizes, possible expert bias, and how well the sample represents the sector should be noted.
- The manuscript has remarkably higher AI usage index, which could be a problem with ethics and may not be within PLOS ONE's permitted range. The authors need to make a lot of changes to the manuscript so that less than 20% of it is flagged by AI and the tone is more scholarly.
- While the manuscript reviews RSU governance practices extensively, it does not clearly differentiate:
  -What this study contributes to current RSU/equity compensation frameworks
  -How the Delphi-AHP hybrid methodology offers innovative perspectives
  -What actionable implications the results have for cross-industry RSU design
- Much of the discussion summarizes prior literature rather than synthesizing new contributions identified through the expert-driven AHP approach and Delphi consensus.
- Introduction:
  -Reviewer 1 correctly notes the need for methodological grounding and updated citations.
  -The last paragraph of the Introduction should clearly show how the manuscript is organized and how the subsequent sections are planned.
- Literature Review:
  -The review must begin with a rationale paragraph framing why a literature review on RSU governance is necessary and proceed with clearer, thematically grouped sources.
  -The current review cites several seminal works but lacks recent evidence from: Behavioral finance on executive incentives, corporate governance reforms post-2020, cross-industry RSU adoption trends etc.
- Add a few recent (preferably 2021 to 2025) and credible sources in citations and references. References should follow APA format and guidelines and also should be arranged in alphabetical order. Further, include the doi link to the resources as much as possible. At least 80% of references must include DOIs where available.
- The manuscript presents both Delphi and AHP methodologies reflecting statistical rigor. Authors should describe the population of specialists, Justify the sample size (n), Explain criteria for picking experts across industries, Clarify whether experts were evenly distributed across sectors
- The manuscript reports two rounds but does not describe: How consensus was measured (e.g., percentage, stability index), How conflicting responses were handled, and the rationale for ending after two rounds

- Give rationale behind “AHP targets by particular industry group”. Also, clarify how to handle missing or incomplete expert responses
- The results section shows weighted RSU governance criteria by industry, but interpretation is limited, making it hard to understand. Also, tables and figures should follow the journal’s guideline.
- References: should follow APA format and guidelines. Pay close attention to the authors cited in the text, as well as the spelling, year of publication, and author verification throughout the text.
- The manuscript shows potential, especially in how it uses Delphi and AHP from different fields to create industry-specific RSU governance structures. But there are some very important issues that need to be addressed, and align with journal standards.

Editorial Decision: ***Major Revision***

Looking forward to receiving a revised version that incorporates these suggestions.

Warm regards,

Academic Editor

PLOS ONE
