# Supplementary material for: Strategic equity compensation: A delphi-AHP approach to industry-specific governance design for RSUs
Source: PLoS One. 2026 Mar 19;21(3):e0343560. doi: 10.1371/journal.pone.0343560 (PMC13001955; doi:10.1371/journal.pone.0343560)
Supplement: Attachment — Submitted filename: Decision Letter to Authors_PONE-D-25-36569R1.pdf [file pone.0343560.s006.pdf]

Decision Letter to Authors

Manuscript ID: PONE-D-25-36569R1
Title: Strategic Equity Compensation: A Delphi–AHP Approach to Industry-Specific RSUs Governance Design

Dear Authors,

Thank you for submitting your updated version of the manuscript to PLOS ONE. I appreciate the time and effort you put into addressing the reviewers' and editorial remarks with clarity and care.

Having reviewed the revised version of your manuscript and careful responses, I am happy to accept your manuscript for publication in *PLOS One*.

Congratulations to you and your co-authors on this achievement, and thank you for choosing *PLOS ONE*.

With best regards,

Academic Editor
PLOS ONE
